# Expression of SPAG7 and its regulatory microRNAs in seminal plasma and seminal plasma-derived extracellular vesicles of patients with subfertility

**DOI:** 10.1038/s41598-023-30744-3

**Published:** 2023-03-04

**Authors:** Masood Abu-Halima, Lea Simone Becker, Mohammad A. Al Smadi, Lea Sophie Kunz, Laura Gröger, Eckart Meese

**Affiliations:** 1 Institute of Human Genetics, Saarland University, 66421 Homburg, Saar Germany; 2 Reproductive Endocrinology and IVF Unit, King Hussein Medical Centre, Amman, Jordan

**Keywords:** Molecular biology, Reproductive disorders

## Abstract

Seminal plasma contains a variety of extracellular vesicles (EVs) that deliver RNAs including microRNAs (miRNAs) molecules. However, the roles of these EVs along with their delivered RNAs and their interactions with male infertility are not clear. Sperm-associated antigen 7 (SPAG 7) is expressed in male germ cells and plays a crucial role in several biological functions associated with sperm production and maturation. In this study, we aimed to identify the post-transcriptional regulation of SPAG7 in seminal plasma (SF-Native) and seminal plasma-derived extracellular vesicles (SF-EVs) collected from 87 men undergoing infertility treatment. Among the multiple binding sites for miRNAs within its 3’UTR of SPAG7, we identified the binding of four miRNAs (miR-15b-5p, miR-195-5p, miR-424-5p, and miR-497-5p) to the 3’UTR of SPAG7 by the dual luciferase assays. Analyzing sperm, we found reduced mRNA expression levels of SPAG7 in SF-EVs and SF-Native samples from oligoasthenozoospermic men. By contrast, two miRNAs (miR-424-5p and miR-497-5p) form the SF-Native samples, and four miRNAs (miR-195-5p, miR-424-5p, miR-497-5p, and miR-6838-5p) from the SF-EVs samples showed significantly higher expression levels in oligoasthenozoospermic men. The expression levels of miRNAs and SPAG7 were significantly correlated with basic semen parameters. These findings contribute significantly to our understanding of regulatory pathways in male fertility by showing a direct link between upregulated miRNA, notably miR-424, and downregulated SPAG7 both in seminal plasma and in plasma-derived EVs likely contributing to oligoasthenozoospermia.

## Introduction

Infertility is a multifactorial reproductive disorder, in which couples are unable to achieve pregnancy within at least one year of regular unprotected sexual intercourse. Approximately 15% of the couples are affected, in which male factors contribute to ~ 50% of the overall infertility cases^1^. Studies have reported many candidate genes indicating or even possibly associated with the regulation of spermatogenesis^2^. Of these genes, some are necessary during sperm maturation and some others provide the milieu required for sperm to gain the ability for the progressive movement and fertilization^2^. However, the functional role and the molecular mechanisms by which each of these genes regulates and maintains sperm production are still poorly understood.

Sperm-associated antigens (SPAGs) are a group of sperm membrane proteins that play a crucial role in several biological functions associated with sperm production and maturation. The expression regulation of SPAGs is important during fertility programs^3^. SPAGs proteins also play a role in the structural integrity of sperm tail, sperm motility, cell adhesion, and cell signaling of sperm and any deficiency of SPAGs proteins may lead to infertility due to sperm dysfunction^4^. Sperm-associated antigen 7 (SPAG7) protein known as FSA1, ACRP, and/or MGC20134^5^ is expressed in male germ cells, specifically during the developing acrosome of round and elongating spermatids, and has been found to trigger the immune response underlying infertility^6, 7^. Like most genes, SPAG7 can be regulated transcriptionally and/or post-transcriptionally, with post-transcriptional regulation relying, among other regulators, on microRNAs (miRNAs). MiRNAs are a class of small single-stranded non-coding RNAs of approximately 22 nucleotides that bind to the 3’untranslated region (3’UTR) of the target messenger RNA (mRNA) resulting in either mRNA degradation and/or repression of protein translation^8, 9^. To date, 2300 miRNAs have been reported as ''true and/or real'' human mature miRNAs^10^. These miRNAs are involved in many, if not all, cellular and biological processes, including reproductive disorders^11–15^ and early stages of embryonic development^16, 17^. It is well-known that one target gene can be regulated by many miRNAs and a miRNA influences the expression of different genes^8^. Therefore, this feature gives miRNAs the capacity to affect the biological functions of different types of cells/tissues. The presence of miRNAs has been confirmed in different cells/tissues and many biological fluids of the male reproductive system, as reviewed by Salas-Huetos et al*.*^18^, indicating their important role during spermatogenesis in sperm production and maturation.

Evidence has begun to accumulate not only for the biological relevance of functional targets of particular miRNAs but also for their potential diagnostic and therapeutic value in male infertility or male contraception^19–25^. Looking at the post-transcriptional regulation level of SPAG7, we found that it has a binding site for seven miRNAs within its 3’UTR, including miR-15a, miR-15b, miR-16, miR-195, miR-424, miR-497, and miR-6838. Interestingly, these miRNAs have been reported to play a functional role in various molecular and cellular stages of germ cell proliferation, differentiation, and features that may have an impact on the maturation of the sperm itself^18^. In seminal plasma, there are many types of extracellular vesicles (EVs) including exosomes that are produced by the luminal prostatic epithelial cells and many other cellular sources in the male genital tract under physiologic and pathologic conditions^26, 27^. These EVs are expelled at ejaculation and likely play a role in the regulation of cellular functions as they contain nucleic acids cargo (DNA, mRNA, microRNA, and other non-coding RNAs)^26, 27^. Thus, by direct transferring their nucleic acid cargoes to target cells, EVs may play important roles in intercellular communication and lead to different biological functions depending on their origin^26^. We performed, differential expression analyses of the SPAG7 target gene and its predicted regulatory miRNAs in seminal plasma (SP-Native) and seminal plasma-derived extracellular vesicles (SP-EVs) collected from men with subfertility and control fertile men. These miRNAs may have a distinct role in seminal plasma and could be used as potential biomarkers for male infertility. In addition, we aim to find out whether their expression levels (i.e., SPAG7 and its seven predicted miRNAs) differ in the SF-Native and SF-EVs. The results of this study provide insights into the potential role of mRNA and its regulatory miRNAs in the regulation of male fertility potential.

## Results

### Basic parameters of fertile and subfertile men

The characteristics of the spermiogram of the subfertile oligoasthenozoospermic men and aged-matched normozoospermic controls are shown in Table 1. Subfertile men with oligoasthenozoospermia were significantly different compared to normozoospermic controls in terms of basic semen parameters (i.e., sperm count, motility, and morphology, *P* < 0.05). Other parameters, such as age, volume, pH, and viscosity, were not significantly different.

**Table 1:** Semen characteristics.

Parameters	Normozoospermic controls	Oligoasthenozoospermic men	*P*-value
Age (year)	35.9 ± 6.70	34.8 ± 7.22	0.510
Volume (ml)	2.98 ± 1.27	3.15 ± 0.76	0.110
pH	8.70 ± 0.35	8.66 ± 0.37	0.573
Count (10^6^/ml)	84.9 ± 14.4	10.0 ± 3.22	0.001
Motility (% motile)	59.6 ± 9.26	18.5 ± 3.21	0.001
Morphology (%)	7.71 ± 2.12	5.69 ± 2.29	0.001

Normozoospermic controls (n = 43) and oligoasthenozoospermic men (n = 44).

An Unpaired two-tailed t-test was used to calculate the *P*-values.

Data were presented as mean ± standard deviation.

*P* < 0.05 was considered statistically significant.

### Validation of miRNA over-expression in HEK-293T

The overexpression of each miRNA was confirmed by RT-qPCR in HEK-293T cells transfected with plasmids overexpressing the seven miRNAs predicted to bind to the 3’UTR of the SPAG7 gene. Transfection resulted in highly increased expression levels of six miRNAs (miR-15b, miR-16, miR-195, miR-424, miR-497, and miR-6838) compared to the controls (pSG5-empty vector) (Supplemental Fig. 1). However, a minimum change in the expression level was observed when pSG5-miR-15a was used to transfect the cells (Fold change 1.4, *P* = 0.032).

### 3’UTR Luciferase reporter assays

**Figure 1:**
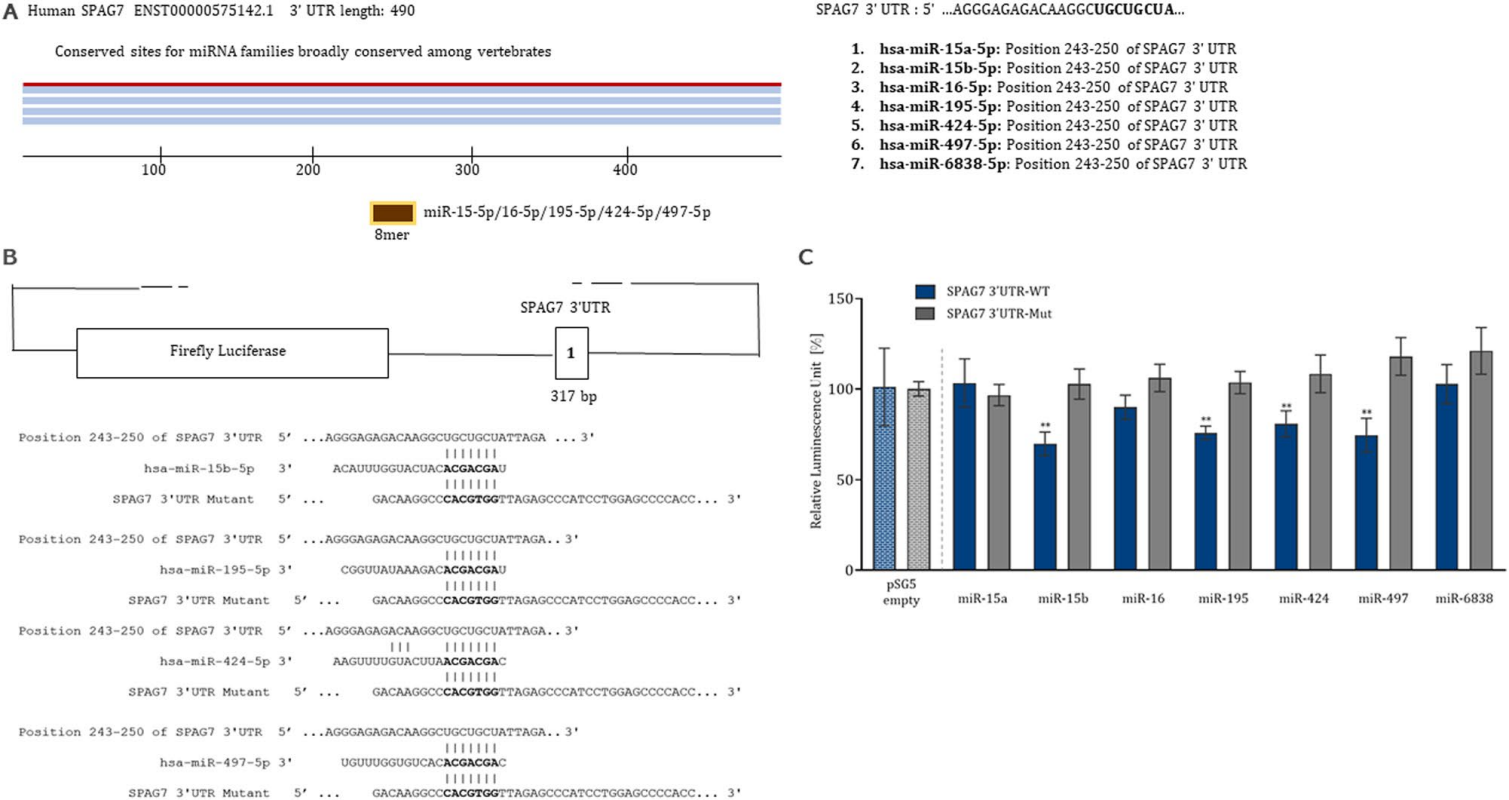
(**A**) Prediction of seven candidate miRNAs potentially binding to the SPAG7 3’-UTR, (**B**) Schematic diagram of the location of the predicted binding sites of miRNAs within the SPAG7 3’UTR region. The sequences of the binding sites of miR-15b, miR-195, miR-424, and miR-497 and the mutated binding sites (bold letters) are shown, (**C**) Dual luciferase assays of the 3’UTRs of SPAG7 and seven tested miRNAs. The results represent the mean of at least three independent experiments carried out in duplicates. Student's t-tests and mean ± SEM were used to evaluate differences in expression level. *P* < .05 was considered statistically significant (*P < 0.05; **P < 0.01; ***P < 0.001).

Using the dual luciferase assay, we examined the regulatory relationship between seven miRNAs and the 3’UTR of the SPAG7 gene. The SPAG7-3’UTR wild-type (WT) and mutant-type (Mut) vectors were constructed and co-transfected with pSG5-miRNA (miR-15a, miR-15b, miR-16, miR-195, miR-424, miR-497, and miR-6838). The location of the targets, the predicted binding sites of seven miRNAs in the 3'UTRs, the sequences of the binding sites, and the mutated binding sites are shown in Fig. 1A,B. The results are expressed as relative luminescence units (RLU) and the ratio between firefly luciferase/renilla luciferase provides the normalized luciferase activity for each tested miRNA. Repression of the luciferase activity as a result of miRNA of interest binding to the 3’UTR of the target gene is shown in Supplemental Fig. 2. Specifically, a significant reduction in luciferase activity was observed when the miR-15b-5p (69.91%), miR-195-5p (75.93%), miR-424-5p (80.82%), and miR-497-5p (74.70%) bind to the SPAG7 3’UTR-WT (Fig. 1C, *P* < 0.001). However, no significant effect on luciferase activity was observed when the four miRNAs bind to the SPAG7 3’UTR-Mut and/or pMIR-RNL-TK empty control vector.

### The expression level of SPAG7 protein in the spermatozoa

**Figure 2:**
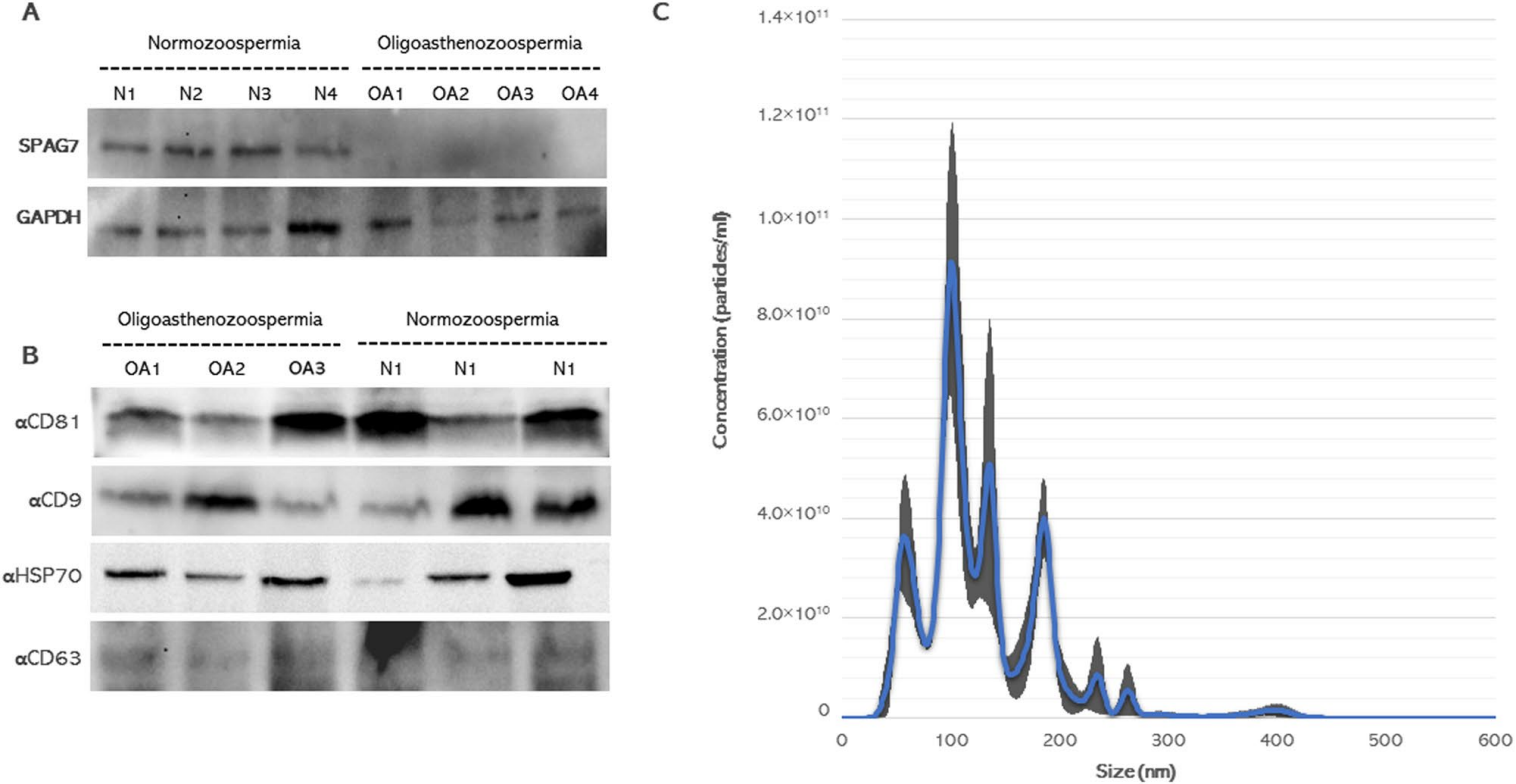
Western blot analysis of (**A**) SPAG7 (~ 26 kDa) and GAPDH (~ 37 kDa) protein levels in the sperm from oligoasthenozoospermic (OA, n = 4) and age-matched normozoospermic men (N, n = 4), (**B**) EVs marker proteins CD81 (~ 26 kDa), CD9 (~ 28 kDa), HSP70 (~ 70 kDa), and CD63 (~ 53 kDa) from sex independent SP-EVs samples, and (**C**) Nanoparticle tracking analysis of particle size and concentration from three independent SP-EVs samples. Mean ± SEM of particle concentration was indicated. Gray is the standard error and the blue line indicates the mean of technical replicates.

To further confirm the results of RT-qPCR, a new set of semen samples collected from oligoasthenozoospermic men (n = 4) and four normozoospermic controls (n = 4) were analyzed by Western blot. Western blot results showed that the trend in expression levels of anti-SPAG7 in sperm is similar to seminal plasma RT-qPCR data. Specifically, sperm samples from oligoasthenozoospermic men showed a reduced level of SPAG7 protein levels compared to control normozoospermic men (Fig. 2A). The average quantified protein level of anti-SPAG7 was 0.88 ± 0.13 in normozoospermic controls *versus* 0.37 ± 0.18 in oligoasthenozoospermic men (*P* = 0.007).

### Detection and characterization of seminal plasma EVs

We purified the EVs from seminal plasma collected from oligoasthenozoospermic men and normozoospermic controls. To confirm the presence of EVs, samples were analyzed for the presence of EVs markers by Western blotting. For this task, proteins were isolated, separated, and probed with antibodies against CD81, CD9, CD63, and HSP70 markers. As shown in Fig. 2B, we detected the CD81, CD9, CD63, and HSP70 proteins, indicating the presence of EVs in the ultracentrifuged pellets. To further confirm the Western blotting analysis, SF-Native seminal fluids were exposed to sequential centrifugation steps and their size and concentration were determined by NTA in an independent set of samples. NTA analysis showed that the SP-EVs collected from three independent samples had an average diameter of 114.5 ± 2.56 nm particles and an average concentration of 5.41 × 10^12^ particles/ml (Fig. 2C). Taken together, these results show that isolated SP-EVs indeed have all EVs characteristics, and that seminal plasma is rich in EVs.

### Detection of mRNA and miRNA expression level

**Figure 3:**
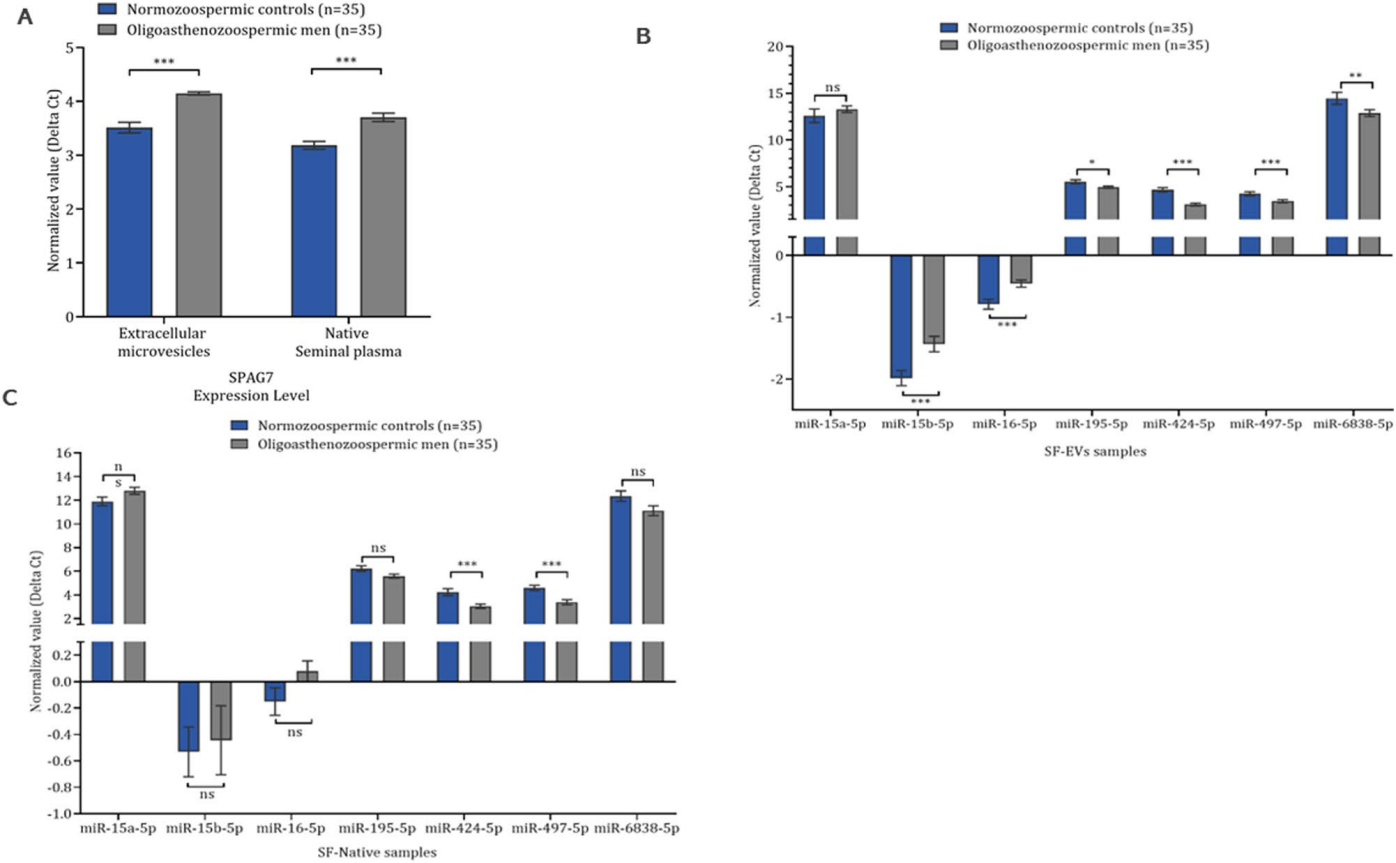
Expression levels of mRNA and seven miRNAs in oligoasthenozoospermic men and age-matched normozoospermic men as determined by RT-qPCR. (**A**) SPAG7 mRNA, (**B**) SF-EVs samples, and (**C**) SF-Native samples. Data are presented as the mean ΔCt of oligoasthenozoospermic and normozoospermic men (lower ΔCt, higher abundance level). GAPDH and global normalization were used as an endogenous control for the normalization of mRNA and miRNAs, respectively. Unpaired-two-tailed t-test and mean ± SEM were used to evaluate differences in expression level. *P* < 0.05 with FDR adjustment was considered statistically significant (*P < 0.05; **P < 0.01; ***P < 0.001).

Differential RT-qPCR was performed to assess the expression levels of SPAG7 and seven miRNAs (miR-15a, miR-15b, miR-16, miR-195, miR-424, miR-497, and miR-6838) in SF-EVs (n = 70) and SF-Native (n = 70) samples collected from the same men with oligoasthenozoospermia (OA, n = 35) and control normozoospermic men (N, n = 35). As shown in Fig. 3A, the expression level of SPAG7 was significantly lower in both samples, in SF-EVs and SF-Native samples collected from oligoasthenozoospermic men compared to normozoospermic controls. The relative expression level was 1.55 and 1.43-fold lower in SF-EVs and SF-Native samples, respectively (*P* = 0.0001 and *P* = 0.0001, respectively), indicating that men with subfertility exhibited a lower expression level of SPAG7 in their purified seminal plasma (i.e., SF-EVs and SF-Native samples).

The expression level of the seven miRNAs was identified in the same samples, which have been used for the SPAG7 expression level analysis [SF-EVs (n = 70) and SF-Native (n = 70) samples]. As shown in Fig. 3B, 6 out of 7 tested miRNAs including miR-15b-5p, miR-16-5p, miR-195-5p, miR-424-5p, miR-497-5p, and miR-6838-5p showed a significant difference in expression level in the SF-EVs samples (*P* < 0.05, FDR adjusted). Out of these 6 miRNAs, 2 miRNAs including miR-15b-5p (fold change = 0.68) and miR-16-5p (fold change = 0.79) showed a significantly lower expression level and 4 miRNAs including miR-195-5p (fold change = 1.49), miR-424-5p (fold change = 2.97), miR-497-5p (fold change = 1.74), and miR-6838-5p (fold change = 3.03) showed a significantly higher expression level in oligoasthenozoospermic men compared to normozoospermic controls.

Similarly, 4 out of 7 tested miRNAs including miR-195-5p, miR-424-5p, miR-497-5p, and miR-6838-5p showed a significant difference in expression level in the SF-Native samples collected from oligoasthenozoospermic men compared to normozoospermic controls (*P* < 0.05). By applying an FDR adjustment for the miRNA that showed a significant change in their expression level, 2 miRNAs including miR-424-5p (fold change = 2.28) and miR-497-5p (fold change = 2.31) were identified with significantly higher expression levels in oligoasthenozoospermic men compared to normozoospermic controls (Fig. 3C, *P* < 0.05, FDR adjusted).

### Correlation of expression levels (ΔCt) of SPAG7 and miRNAs with basic semen parameters

We next analyze correlations between the expression level of SPAG7 and miRNAs in seminal plasm and the basic semen parameters i.e., sperm count, motility, and morphology. Spearman correlation analysis showed weak, moderate to strong correlations between SPAG7, miRNAs, and sperm count, motility, and morphology (Supplementary Table 4, *P* < 0.05). Specifically, spearman correlation analysis showed a significant positive correlation between the expression level of SF-native SPAG7 and basic semen parameters whereas significant negative correlation between the SF-native miR-424-5p and the basic semen parameters (Table 2). Furthermore, a negative correlation between the lower expression level of SF-native SPAG7 genes and the higher expression level of SF-native miR-424-5p was observed in oligoasthenozoospermic men compared to normozoospermic controls. Significant weak correlations between the expression level of other tested SF-native miRNAs, SPAG7, and semen parameters were identified.

**Table 2:** Correlation analysis between the expression level of miRNAs, SPAG7, and basic semen parameters.

Parameters		Count (10^6^/ml)	Motility (% motile)	Morphology (%)	SPAG7
miR-424-5p	Correlation Coefficient	− 0.526	− 0.602	− 0.274	− 0.406
	*P* value	0.001	0.001	0.024	0.001

Parameters		Count (10^6^/ml)	Motility (% motile)	Morphology (%)	miR-424-5p
SPAG7	Correlation coefficient	0.576	0.611	0.238	− 0.406
	*P* value	0.001	0001	0.048	0.001

Spearman correlation analysis.

An Unpaired two-tailed t-test was used to calculate the *P*-values.

*P* < 0.05 was considered statistically significant.

## Discussion

In this study, the presence of EVs in the seminal plasma samples collected from men undergoing infertility treatment was verified by Western blotting and NTA analyses. EVs purified from human seminal plasma showed an average size of 114.5 ± 2.56 nm, which are typical EV features, and an average particle concentration of 5.41 × 10^12^ particles/ml. As an independent technique, Western blotting analysis was carried out and showed that the SP-EVs expressed the EVs markers CD81, CD9, CD63, and HSP70. EVs originate from different cells and are released abundantly, not only in seminal plasma but also in nearly all biological fluids^28^. EVs modulate intercellular communication between tissues and/or cells by packaging and transporting different functional elements including nucleic acid cargo to distant sites^29^. Therefore, identifying the expression level of a certain mRNA(s) or a miRNA(s) in SP-EVs may help to provide more insights into the potential role of miRNAs in the regulation of target genes in male infertility. In our study, as shown in Table 1, there was a statistically distinguishable difference between the oligoasthenozoospermic and normozoospermic men based on sperm count. However, there was no significant difference in the concentration and the average size of EVs between them (i.e., oligoasthenozoospermic and normozoospermic men), indicating that semen contains a variety of EVs, which are released from different parts of the male reproductive system regardless of sperm count. The different classes of EVs play a major role in sperm functions like maintaining sperm motility and survival capacity in the female reproductive tract and spermatogenesis^30^.

Although differential miRNA expression levels in SP-EVs and SP-Native have been reported in previous studies on male infertility^15, 31–33^, there are only a few examples of a well-documented functional link between a deregulated miRNA and a target gene in spermatogenesis and spermatogenesis-related cells^19, 20, 22–25^. Here, the expression levels of miR-15a-5p, miR-15b-5p, miR-16-5p, miR-195-5p, miR-424-5p, miR-497-5p, miR-6838-5p and that of SPAG7 mRNA were determined in both, in SF-EVs and SF-Native samples, collected from men undergoing infertility treatment. Additionally, the SPAG7 protein expression level was confirmed to exhibit a lower expression level in sperm samples. In contrast, a higher expression level of miR-195-5p, miR-424-5p, miR-497-5p, and miR-6838-5p was observed in oligoasthenozoospermic men compared to normozoospermic controls. No comparable downregulation was found for miR-15a-5p, miR-15b-5p, and miR-16-5p, although all seven miRNAs have the same seed sequence and/or the same binding site within the 3’UTR region of the SPAG7. Nevertheless, the latter miRNAs may have a post-transcriptional impact via inhibition of the protein translation without having an effect at the mRNA level^34^. It is possible, however, that there are other sequences besides the seed sequence, which might be responsible for regulation. An influence of the target site architecture, i.e., the spatial arrangement of the region surrounding the binding site, can be excluded, since the same target gene, SPAG7, was always studied^35^. Similarly, an inverse direction of regulation was observed between the expression level of miRNAs and basic semen parameters, suggesting an association between higher and/or lower expression levels of miRNAs and/or SPAG7 in men with subfertility, probably through influencing sperm count, motility, and morphology. In line with the idea that miRNAs are loaded from the cells of the genital tract into EVs, we found miR-424-5p and miR-497-5p both in SF-EVs and SF-Native. Many other miRNAs were only differentially expressed in SF-EVs (miR-15b-5p, miR-16-5p, miR-195-5p, and miR-6838-5p) (Fig. 3). This result is in agreement with our previous findings that the SF-EVs fraction has a distinct composition of miRNAs that is common to extracellular vesicles collected from seminal plasma^15^. Moreover, the distinct composition of miRNAs can also be influenced by the number of germ cells produced in the testis and mirrors to a large extent the miRNA pattern in spermatozoa^13, 15^. This distinct miRNA portrait contains many potential candidate biomarkers that can be used for the diagnosis and pursued in future clinical studies.

Not much is known about SPAG7 function, but it is thought to be associated with pathological conditions in humans^36^. For example, SPAG7 is associated with PFAPA (Periodic Fever, Aphthous Stomatitis, Pharyngitis, Adenitis) syndrome, an autoinflammatory disorder, and with Asperger syndrome^37, 38^. It is also thought to play a role in the reaction of viruses or free DNA and RNA, as it has a R3H domain that can bind ssDNA or ssRNA in a sequence-specific manner^37^. Based on the Human Protein Atlas, SPAG7 protein is expressed in the testis, epididymis, seminal plasma-derived vesicles, and prostate, as well as in other tissues such as muscle, adipose, and nerve. More specifically, the SPAG7 protein is located in the inner acrosome compartment of sperm and is highly expressed in the round and elongated spermatids^6^. Like most genes, SPAG7 is likely to be post-transcriptionally regulated by miRNAs. Our PCR data indicate miR-195-5p, miR-424-5p, miR-497-5p, and miR-6838-5p as potential regulators, and our combined RT-qPCR and Luciferase data indicate miR-195-5p, miR-424-5p, and miR-497-5p as regulators of the SPAG7 mRNA. Since the protein is expressed on the developing acrosome, the absence of the protein leads to improper development of the acrosome and/or disrupts acrosome formation^6^. It is legitimate to hypothesize that aberrations in miR-195-5p, miR-424-5p, and miR-497-5p expression levels can adversely influence SPAG7 during the spermatogenesis process and might reduce fertility potential. The acrosome and its acrosome reaction are required for successful fertilization^39^. Taken together, these results suggest that the lower expression level of SPAG7 in either SP-EVs and/or SF-Native could have an adverse effect on male fecundity.

The role of miRNAs during testicular development and spermatogenesis of mammals has been studied for several years and these studies confirmed that altered expression of miRNA affects the production and/or the function of sperm, as reviewed by Salas-Huetos et al*.*^18^. It is interesting to find out that the miR-15a, miR-15b, and miR-16 are expressed at a lower level in SP-EVs in oligoasthenozoospermic men compared with normozoospermic men. This result is in agreement with other studies, in which miR-15a, miR-15b, and the miR-16 expression level were reduced in sperm of men with subfertility and varicocele-related sperm impairment^13, 22^, in SP-EVs of men with subfertility^15^, and in testicular tissues of men with a different pattern of non-obstructive azoospermia^11^. Alteration of expression of these miRNAs (i.e., miR-15a, miR-15b, and miR-16) might have a negative impact on male fertility potential. An effect of the lower expression of these three miRNAs on SPAG7 mRNA is unlikely, although they may well possess a post-transcriptional effect in terms of inhibition of protein translation. It is possible that these miRNAs concurrently target functionally related genes to drive a specific biological effect associated with male infertility. For example, miR-15a targets the cyclin T2, which is involved in the early stages of spermatogenesis^40^, while miR-15b targets the mRNA encoding isocitrate dehydrogenase 3 alpha (IDH3A) and regulates TCA-cycle mediated energy metabolism^41^. The reduced expression level of IDH3A in turn interferes with sperm motility by changing energy metabolism^41^. Additionally, the other miRNAs i.e., miR-195-5p, miR-424-5p, miR-497-5p, and miR-6838-5p may also participate in reducing male fertility and may play a role in many other physiologic processes that are related to male infertility. Many supporting lines of evidence have indicated that miR-195-5p functions as a tumor suppressor in different types of cancer, including prostate cancer^42–45^. Moreover, Mahn et al*.* found a higher expression level of miR-195 in serum samples collected from patients with prostate cancer^46^. Similarly, miR-424-5p and miR-497-5p play a crucial role in the progression of prostate cancer^47–50^ and miR-6838-5p enhances the proliferation and invasion of renal cell carcinoma^51^. Nevertheless, the way how miR-195-5p, miR-424-5p, miR-497-5p, and miR-6838-5p are delivered to SP-EVs and/or SP-Native and how these miRNAs regulate, not only SPAG7 but also other target genes related to spermatogenesis warrants further study.

In summary, the expression level of the SPAG7 gene and its regulatory miRNAs was carried out on seminal plasma and seminal plasma-derived EVs collected from men with subfertility and fertile controls. Lower expression levels of the SPAG7 gene and miR-15a, miR-15b, and miR-16, and higher expression levels of miR-195, miR-424, miR-497, and miR-6838, were identified. These findings indicate a functional link between miR-195, miR-424, and miR-497, and the SPAG7 gene expression. The miRNA-target gene interaction causes down-regulation of the SPAG7 gene, which may be associated with infertility. On the other hand, miR-15a, miR-15b, and miR-16 were excluded as potential regulators of SPAG7 based on the RT-qPCR data, but this does not mean that these miRNAs might not still have a post-transcriptional influence on SPAG7 through inhibition of protein translation. The results of the dual luciferase assay revealed that miR-195, miR-424, and miR-497 are potential regulators of the SPAG7 gene. These findings contribute in several ways to our understanding of the potential role of miRNAs in the regulation of male fertility.

## Methods

### Patients and samples collection

The study was approved by the Ethics Committees of Saarland/Germany and Saarland University [Institutional Review Board (Ha95/11-2021, updated), and informed written consent was obtained from all patients. The methods in this study were carried out in accordance with the approved guidelines by the University Hospital of Saarland and all experimental protocols were approved by the ethics committee. Semen samples were collected from 87 male partners of couples undergoing IVF treatment, including oligoasthenozoospermic men (n = 43) and age-matched normozoospermic control men (n = 44). These samples were classified as seminal plasma samples from oligoasthenozoospermic men (n = 35) and age-matched normozoospermic control men (n = 35) and sperm samples from oligoasthenozoospermic men (n = 4) and age-matched normozoospermic control men (n = 4). Additionally, 9 semen samples were used for the purification and confirmation of EVs including oligoasthenozoospermic men (n = 4) and normozoospermic control men (n = 5). The clinical characteristics of the patients and controls are summarized in Table 1. Oligoasthenozoospermic men were not presented with any known clinical factors for infertility, such as anatomic malformations, genetic abnormalities, varicocele, and reproductive tract infections, and were not exposed directly to environmental contaminants. Age-matched normozoospermic control men were included among healthy normozoospermic men with no infertility diagnosis and with clearly apparent female factors as a possible cause of the couple's inability to conceive.

After at least three days of sexual abstinence, semen samples were collected, liquefied at 37 °C for 30 min, and evaluated according to World Health Organization 2010 (WHO) guidelines for the basic semen parameters such as volume, pH, viscosity, motility, viability, and morphology. Semen samples were then applied to discontinuous 45–90% PureSperm^®^ 100 Gradient (Nidacon) and centrifuged at 500 × g for 20 min at room temperature (RT). The supernatant containing only the seminal plasma was removed and transferred into a sterile Eppendorf-1.5 ml tube and stored at − 80 °C until EVs and RNA including miRNAs isolation. As for the sperm samples, sperm was collected and a discontinuous PureSperm^®^ density gradient was performed as previously described^19, 20^.

### Prediction of miRNAs targeting SPAG7

TargetScan 7.2 (www.targetscan.org)^52^, was used to predict the miRNAs that exhibited a binding site within the 3’UTR region of the SPAG7 target gene, resulting in the detection of seven miRNAs including miR-15a, miR-15b, miR-16, miR-195, miR-424, miR-497, and miR-6838 (Fig. 1A).

### Plasmid constructs

Plasmids were constructed by cloning the miR-15a, miR-15b, miR-16, miR-195, miR-424, miR-497, and miR-6838 precursors downstream to the T7 viral promotor of the pSG5 expression vector (Agilent Technologies). The synthesized fragments were delivered as inserts and the restriction enzymes *EcoRI* and *BamHI* (New England Biolabs) were used to subclone the fragments into the pSG5 vector. In addition, the 3’UTR of SPAG7 was cloned into the pMIR-RNL-TK dual-luciferase reporter gene vector (Thermo Fisher Scientific), downstream to the luciferase gene using *SpeI* and *SacI* restriction enzymes (New England Biolabs). With this construct, we determined the ability of miRNAs to suppress luciferase expression when it binds to the 3’UTR of the target SPAG7. SPAG7-3’UTR insert was amplified using cDNA prepared from human testicular tissue RNA. Plasmids containing the wild-type 3’UTR are designated with the suffix-WT, and those containing the 3’UTR with mutated miRNA binding sites are designated with the suffix-Mut. As for the mutated binding site of the 3’UTR of SPAG7, we changed the binding properties of the miRNA binding site with the PmlI restriction enzyme (New England Biolabs) sequence to make sure that the miRNA will not bind. Specific primer sequences and restriction sites are listed in Supplemental Table 1 and the size, reference, and location of the cloned fragments are listed in Supplemental Table 2. The precursor sequences of the miRNAs with EcoRI and BamHI cleavage sites are listed in Supplemental Table 3.

### Cell lines and cell culture

The human embryonic kidney cell line (HEK-293T) was purchased from the German collection of microorganisms and cell cultures (DSMZ) and used for the dual-luciferase reporter assays and transfection procedures. Both the reporter plasmid (pMIR-RNL-TK) and the expression plasmid (pSG5) harbor the SV40-promotor. The cells were maintained in Dulbecco’s modified Eagle’s medium (DMEM) (Thermo Fisher Scientific) supplemented with 10% Fetal Bovine Serum (FBS) (Biochrom GmbH), 100 U/mL Penicillin, and 100 μg/mL Streptomycin (Sigma–Aldrich). Cells were incubated at 37 °C in a humidified 5% CO2 atmosphere and subcultured following trypsinization with a 1X solution of 0.05% Trypsin–EDTA (Thermo Fisher Scientific).

### Overexpression of miRNAs by using expression plasmids in HEK-293T cells

HEK-293T cells (4 × 10^5^ cells) were seeded in 6-well plates and transfected with a reporter plasmid (pMIR-RNL-TK) overexpressing SPAG7. After 24 h, cells were transfected with 20 µl of PolyFect™ Transfection Reagent (Qiagen) and incubated at 37 °C and 5% CO2 for 48 h. Following incubation, RNA was isolated using the miRNeasy^®^ Mini Kit, cDNA was generated using TaqMan^®^ MicroRNA Reverse Transcription Kit, and RT-qPCR was performed using TaqMan^®^ Small RNA Assay for each miRNA assay. All RT-qPCR reactions were set up using a QIAgility automated PCR setup robot (Qiagen) and detected using StepOnePlus™ Real-Time PCR System.

### Transfection and 3’UTR luciferase reporter assays

Using the automatic liquid handling system epMotion 5075 (Eppendorf) along with Dual Luciferase^®^ Reporter-Assay kits (Promega), HEK-293T cells (3.8 × 10^4^ cells) were seeded in 96-well culture plates. Twenty-four hours later, cells were transfected with 0.05 µg of the corresponding reporter gene construct pMIR-empty and either 0.2 µg of pSG5 or a corresponding miRNA, or they were transfected with 0.05 µg of pMIR-SPAG7 and 0.2 µg of pSG5 or a corresponding miRNA. Every transfection was performed three times for each of the 7 miRNAs (pSG5-miR-15a, pSG5-miR-15b, pSG5-miR-16, pSG5-miR-195, pSG5-miR-424, pSG5-miR-497, and pSG5-miR-6838). Following the manufacturer's recommendations, 1 µl PolyFect™ Transfection Reagent (Qiagen) was used to transfect the cells, which were then incubated at 37 °C and 5% CO2 for 48 h. The Luciferase Assay was determined automatically and evaluated using the GloMax^®^ Navigator microplate luminometer (Promega). Results were expressed as mean relative luciferase activity ± standard error (Firefly luciferase light units/Renilla luciferase light units).

### Protein isolation and western blotting for sperm samples

A western blot experiment was carried out to identify the SPAG7 protein expression level in sperm samples. Semen samples were obtained from an independent cohort of oligoasthenozoospermic men (n = 4) who attended the IVF center for infertility treatment and normozoospermic men served as controls (n = 4). Samples were thawed on ice and washed three times with PBS. The samples were then centrifuged at 14,000 × g at 4 °C for 15 min. The pellet was suspended in 100 µl RIPA buffer (Thermo Fisher Scientific) supplemented with a protease inhibitor (Sigma–Aldrich). Then, the samples were sonicated at 20 J for 2 s × 10 at intervals of 10 s and incubated on ice overnight inside the fridge at 4 °C. The next day, samples were centrifuged at 4 °C for 10 min at 14,000 × g, supernatant from each sample was transferred into a new tube, and protein concentrations were quantified using the BCA protein assay kit (Pierce). Thirty micrograms of total protein from each sample were denatured with Laemmli buffer mixed with b-mercaptoethanol (1:4; Bio-Rad Laboratories) and heated at 95 °C for 5 min. Anti-SPAG7 antibody [EPR13391, Abcam] diluted in TBS Blotto A (1:1000) and GAPDH (14C10) rabbit mAb antibody (1:1000, 2118S, Cell Signaling Technology) were used to detect the protein expression level. Each primary antibody was incubated individually and precision plus protein standard (Bio-Rad Laboratories) was used for the size determination. Following incubation with an anti-rabbit secondary antibody (1:3,000, A0545, Sigma-Aldrich), the gray value of each protein band was analyzed on a ChemiDoc™ Touch system (Bio-Rad Laboratories) and normalized to that of GAPDH.

### Enrichment of EVs from seminal plasma

The enrichment of EVs was performed by sequential centrifugation steps. Briefly, seminal plasma samples were centrifuged at 4 °C for 10 min at 1600 × g, and the supernatant was transferred into a new tube, and centrifuged once again at 4 °C for 10 min at 16,000 × g. The collected supernatant was treated with 1% v/v Triton X-100 (Sigma-Aldrich) and incubated on ice for 20 min. Then, each sample was divided into two parts, one part (250 µl) was used to isolate EVs by ultracentrifugation (and subsequently protein and RNAs isolation) and the second part (250 µl) was used to isolate the RNAs including miRNA from the native samples (SF-Native). After ultracentrifugation at 100,000 × g for two hours at 4 °C, two fractions were obtained, the enriched EVs and the EV-depleted seminal plasma fraction. The EV pellets were collected resuspended in PBS and were used to purify the proteins and RNAs including miRNAs.

### Total RNA, including miRNAs purification

For RNA including miRNA purifications, SF-EVs (n = 70) and SF-Native (n = 70) samples were mixed with 600 µl QIAzol Lysis Reagent (Qiagen) and 100 µl Dithiothreitol (0.1 M, DTT) (Sigma–Aldrich). Samples were incubated for 5 min at RT and then 140 µl of chloroform was added, vortexed, and incubated for an additional 3 min at RT. The mixture was then centrifuged at 4 °C for 20 min at 14,000 × g, 12 µl glycogen (5 ng/µl) (Thermo Fisher Scientific) was added to each collected supernatant, and miRNeasy^®^ Mini (Qiagen) Kit was used to purify the RNA including miRNAs. Elution was performed with 15 µl of RNase-free water and quantification was performed using NanoDrop™ 2000c Spectrophotometers (Thermo Fisher Scientific). RNA quality was randomly checked using a Bioanalyzer 2100 instrument (Agilent Technologies).

### Protein isolation and Western blotting for EVs samples

As for the protein purification from the SF-EVs, 100 µl of RIPA buffer (Thermo Fisher Scientific) supplemented with 1 × protease inhibitor cocktail (Sigma–Aldrich) was added to each pellet after ultracentrifugation. Samples were incubated on ice for 30 min and then centrifuged at 4 °C for 10 min at 14,000 × g. The supernatants were transferred into new tubes and the protein concentration for each sample was quantified using the BCA protein assay kit (Pierce). After protein isolation and quantification, 30 µg of protein lysate was mixed with Laemmli buffer (3:1 ratio) (Bio-Rad Laboratories) and heated at 95 °C for 5 min. Proteins were then separated by 10% standard sodium dodecyl sulfate–polyacrylamide gel electrophoresis (SDS-PAGE) and transferred to polyvinylidene difluoride (PVDF) membranes (Bio-Rad Laboratories). Blocking was performed with TBS Blotto A (Santa Cruz Biotechnology) for one hour and primary antibodies directed against EVs marker proteins including CD63, CD9, HSP70, and CD81 (System Biosciences) were incubated in a dilution of 1:1000 in TBS Blotto A overnight. Each primary antibody was incubated individually and precision plus protein standard (Bio-Rad Laboratories) was used for the size determination. The membranes were washed three times with 1X TBS-T, each for 15 min before one-hour incubation with goat-anti rabbit HRP antibody diluted 1:20,000 in TBS Blotto A (System Biosciences). After incubation, the membranes were washed another three times, each for 15 min and detection was performed using an enhanced chemiluminescence substrate (Cell Signaling Technology) on the ChemiDoc™ Touch system.

### Nanoparticle tracking analysis (NTA)

The SF-EVs particle size and concentration were measured using NanoSight (Malvern Panalytical). After ultracentrifugation, the pellets containing the SF-EVs were resuspended in 600 µl of filtered PBS. Samples were further diluted at 1:2000 or 1:3000 with PBS and 300 µl of diluted SF-EVs were introduced into the measuring chamber. Next, a video was recorded with a camera level of 15. Each sample was recorded 3 times for 30 s. Particle concentration and size were calculated by NTA 3.4 software with a detection threshold set to 5.

### Validation by RT-qPCR of SPAG7 and miRNAs

RT-qPCR was performed to determine the expression level of SPAG7 and 7 miRNAs in SF-EVs (n = 70) and SF-Native (n = 70) samples. As for the SPAG7 expression level, the High-Capacity RNA-to-cDNA™ and TaqMan™ Fast Advanced Master Mix kits (Thermo Fisher Scientific) along with the StepOnePlus™ Real-Time PCR system, were used. All steps were carried out according to the manufacturer's recommendations. Briefly, 60 ng RNA was used as a template for cDNA synthesis, and primer assays including SPAG7 (Hs00959646_g1) and GAPDH (Hs02786624_g1) (Thermo Fisher Scientific) were used to amplify the generated cDNA for each included sample. All RT-qPCR reactions were set up using a QIAgility automated PCR setup robot (Qiagen).

As for the miRNA analysis, TaqMan^®^ MicroRNA Reverse Transcription Kit and RT Primer Pools (10×) (Thermo Fisher Scientific) along with Biomark™ HD and 96.96 IFC (Fluidigm Corporation) were used as previously described^53, 54^. Briefly, 60 ng of RNA, including miRNAs were reverse transcribed into cDNA. The generated cDNA was preamplified using the TaqMan™ PreAmp Master Mix (2X) and the PreAmp Primers Pool (10×). Lastly, RT-qPCR was performed using 96.96 Dynamic Array™ IFC (Fluidigm). For every 10× Assay, 3 µl TaqMan Primer Assay (20×) (Thermo Fisher Scientific) and 3 µl Assay Loading Reagent (2X) (Fluidigm) were mixed and a Sample Pre-Mix was prepared by combining 3 μl TaqMan™ Universal PCR Master Mix, no AmpErase™ UNG (2X) (Thermo Fisher Scientific), 0.3 μl GE Sample Loading Reagent (20×) (Fluidigm) and 2.7 μl pre-amplified cDNA for each sample. The array was loaded such that 5 µl of the Assay Mix and 5 µl of the Sample mixture were added to each inlet and placed in the Biomark HD instrument. For quantification, the GT 96 × 96 Standard v1 PCR thermal protocol was used.

### Statistical analysis

The statistical analysis was performed using GraphPad Prism Software version 7 (GraphPad Software). Clinical variables are presented as mean ± standard deviation and/or standard error, as appropriate. A priori power analysis with α and β error probability of 0.05 was applied to determine the sample size for each group, showing that ≥ 27 samples per group were required. Direct comparisons between paired data sets were performed using Student's t-test, and the unpaired two-tailed t-test was used to evaluate the differences in miRNA and mRNA expression levels between subfertile and fertile men. The ∆Ct (cycle threshold) was used to measure the dynamic change of the target SPAG7 mRNA and its regulatory seven miRNAs using GAPDH and global normalization as endogenous control, respectively. In our study, the expression level of 12 out of 25 miRNAs showed a stable and non-significant expression level in both fertile and subfertile samples that were used for global normalization. Spearman's correlation was used to measure the association between semen parameters and expression levels of the target SPAG7 mRNA and 7 miRNAs. The differentially expressed mRNA and miRNAs were considered statistically significant if they exhibited an adjusted *P* < 0.05 after applying the multiple comparisons using Benjamini–Hochberg procedure. Quantification of SPAG7 western blot was performed using the Image Lab 6.1 Software (Bio-Rad Laboratories). The signal intensities of bands were determined and normalized with the GAPDH of each sample. Mean ± standard deviation of normalized signal intensities was generated.

### Ethics approval

Institutional Review Board approval/Ethikvotum Arztekammer des Saarlandes: Ethical vote No. Ha95/11-2021, updated.

## Supplementary Information

Supplementary Information.
