# Supplementary material for: Expression of SPAG7 and its regulatory microRNAs in seminal plasma and seminal plasma-derived extracellular vesicles of patients with subfertility
Source: Sci Rep. 2023 Mar 4;13:3645. doi: 10.1038/s41598-023-30744-3 (PMC9985644; doi:10.1038/s41598-023-30744-3)
Supplement: Supplementary file 1 — Supplementary Information. [file 41598_2023_30744_MOESM1_ESM.pdf]

Expression of SPAG7 and its regulatory microRNAs in seminal plasma and seminal plasma-derived extracellular vesicles of patients with subfertility

Masood Abu-Halima [1*♠], Lea Simone Becker [1♠], Mohammad A. Al Smadi [2], Lea Sophie Kunz [1], Laura Gröger [1], and Eckart Meese[1]

[1] Institute of Human Genetics, Saarland University, 66421 Homburg/Saar, Germany.

[2] Reproductive Endocrinology and IVF Unit, King Hussein Medical Centre, Amman, Jordan.

*Correspondence: masood@daad-alumni.de

♠The authors contributed equally to this work

Supplementary information

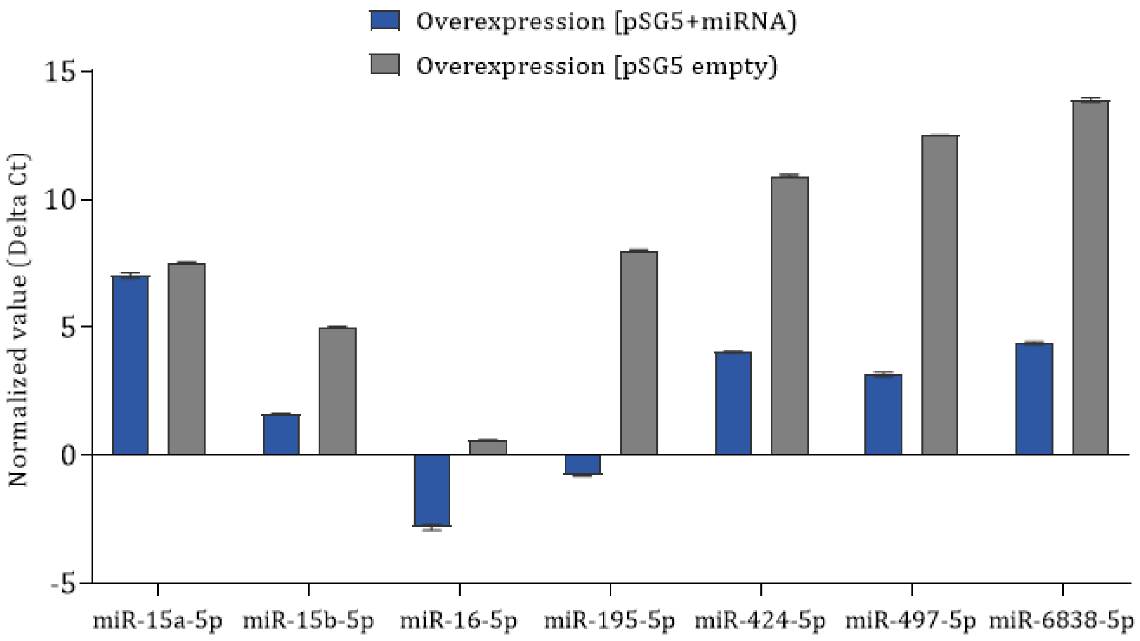

Supplemental Figure 1: Overexpression of miRNAs in HEK-293T cells using the cloned expression plasmids transfection as determined by RT-qPCR.

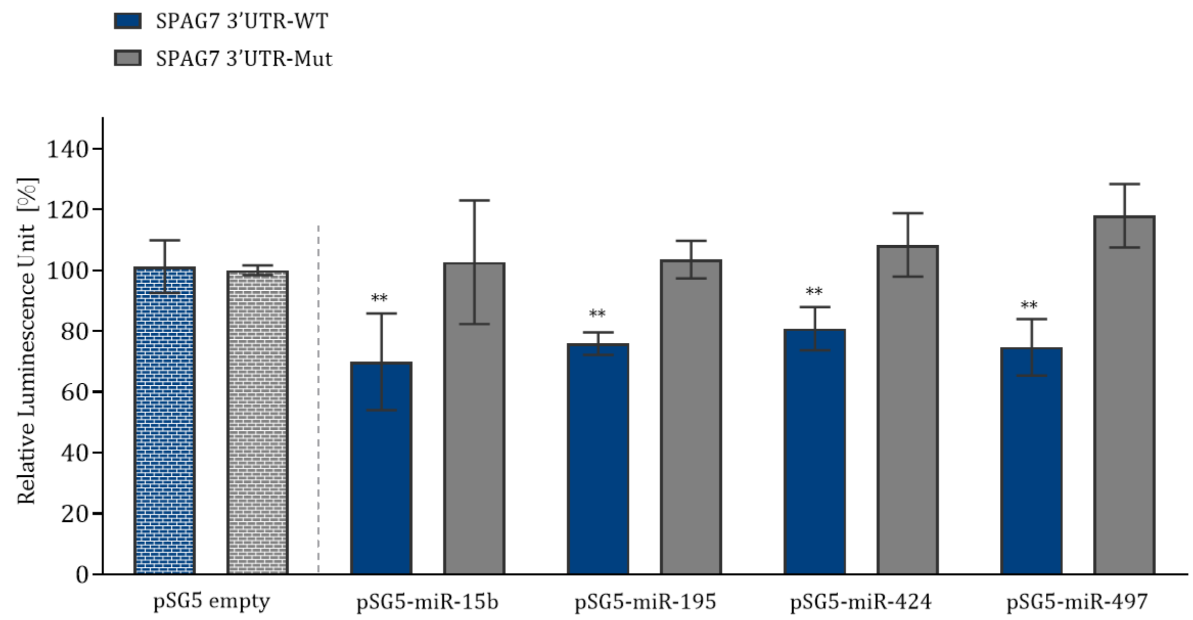

Supplementary Figure 2: Dual luciferase assays of the 3’UTRs of SPAG7 and significant miRNAs. The results represent the mean of at least three independent experiments carried out in duplicates. Student's t-tests and mean ± SEM were used to evaluate differences in expression level. P<.05 was considered statistically significant (*P<0.05; **P<0.01; ***P<0.001).

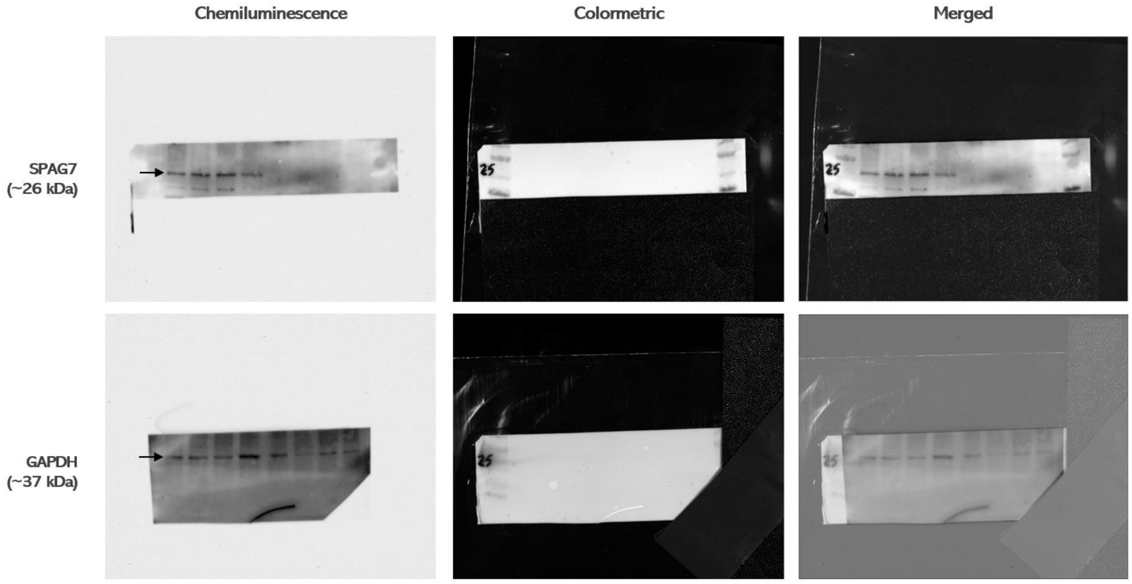

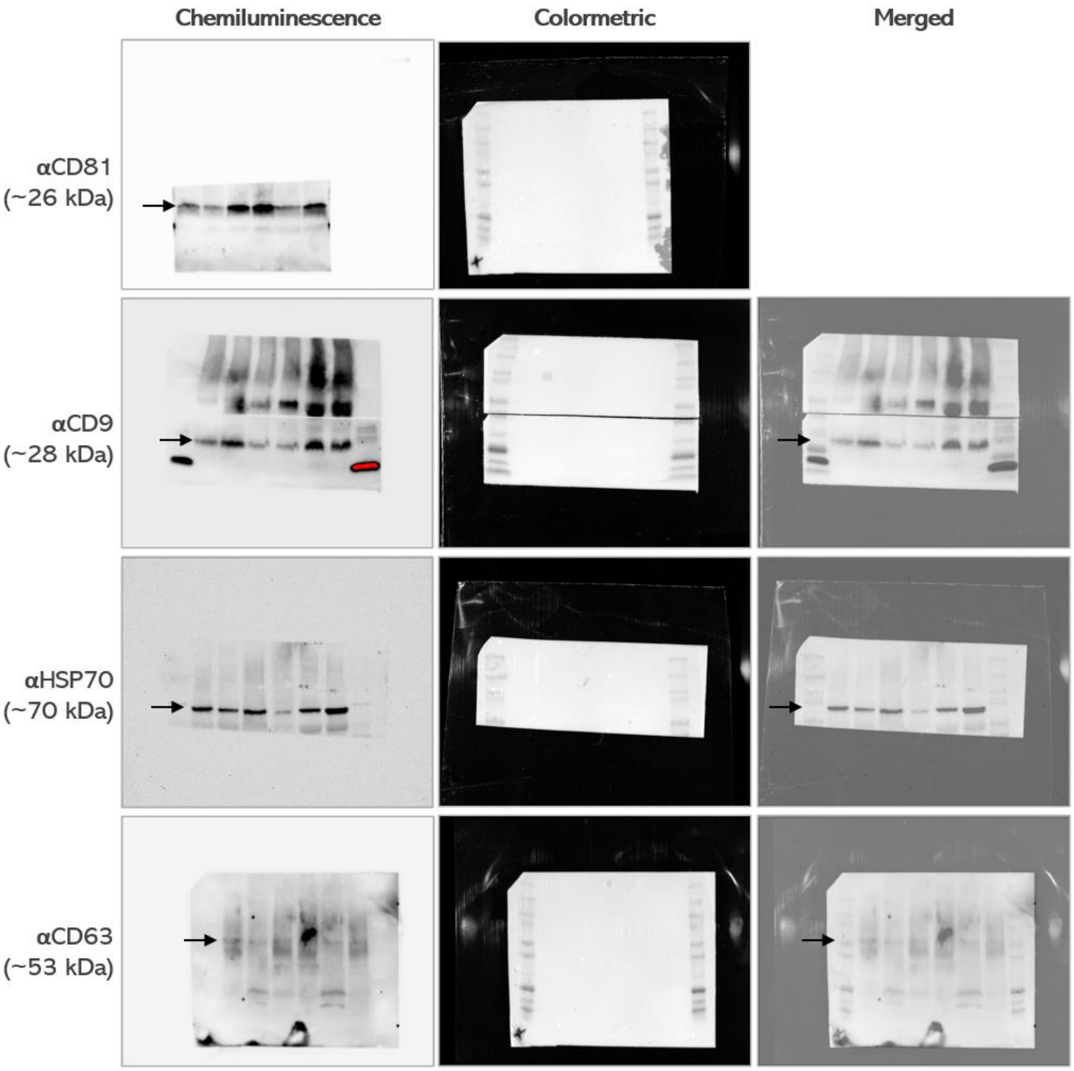

Supplementary Figure 3: Western blot analysis of SPAG7 (~26 kDa) and GAPDH (~37 kDa) protein levels in the sperm from oligoasthenozoospermic (n =4) and age-matched normozoospermic men (n = 4), EVs marker proteins CD81 (~26 kDa), CD9 (~28 kDa), HSP70 (~ 70 kDa), and CD63 (~ 53 kDa) from sex independent SP-EVs samples

Supplementary Table 1: Sequences of cloning and mutagenesis primers

Gene	Sequence
	(Restriction sites are in bold and mutated sites are shown in bold, and highlighted in gray)
SPAG7_For (SpeI)	GG**ACTAGT**CAGAGTGGGGAAGAGTTGCC
SPAG7_Rev (SacI)	CG**AGCTC**TGTAAAGTACCCCAGCCCCA
SPAG7 Mut_For (PmlI)	GACAAGGCC**CACGTG**GTTAGAGCCCATCCTGGAGCCCCACC
SPAG7 Mut_Rev	GGTGGGGCTCCAGGATGGGCTCTAACCACGTGGGCCTTGTC

Supplementary Table 2: Size and reference and location of the cloned fragments

Construct	Sense primer	Antisense primer	Size of amplified fragment	Reference	Nucleotide position
**pMIR-SPAG7**	SPAG7-*Spe*I	SPAG7-*Sac*I	317 bp	NM_004890.3	Coding sequence (CDS) 649-965

Supplementary Table 3: Precursor sequence of the individual microRNAs with EcoRI and BamHI cleavage sites

miRNA	Assay ID TaqMan® Assays	Insert	**Precursor sequence** (grey) **with EcoRI and BamHI sites** (bold)
hsa-miR-15a	ID: 000389	424 bp	**GAATTC**CACTGTGCTGGGCACAGAATGGACTTCAGTTAAGTTTTTGATGTAG<br>AAATGTTTTATTATTCTACTTAAAATCTCCTTAAAAATAATTATGCATATTACAT<br>CAATGTTATAATGTTTAAACATAGATTTTTTTACATGCATTCTTTTTTTCCTGAA<br>AGAAAATATTTTTTATATTCTTTAGGCGCGAATGTGTGTTTAAAAAAAAATAAAA<br>CCTTGGAGTAAAGTAGCAGCACATAATGGTTTGTGGATTTTGAAAAGGTGCA<br>GGCCATATTGTGCTGCCTCAAAAATACAAGGATCTGATCTTCTGAAGAAAAT<br>ATATTTCTTTTTATTCATAGCTCTTATGATAGCAATGTCAGCAGTGCCTTAGC<br>AGCACGTAAATATTGGCGTTAAGATTCTAAAATTATCTCCAGTATTAACTGTG<br>CTGC**GGATCCC**
hsa-miR-15b	ID: 000390	379 bp	**GAATTC**GCCATGGAATTGACTTGGACCATAATAGATTTTTAATTTTACAGGTA<br>AGTTTATTAAAGACTTCAAAGATTCTCTTATTCTTGTTACTTTTTTTTCTATAAA<br>GCTAGGTTGGATGAATCCTACATTTTTGAGGCCTTAAAGTACTGTAGCAGCA<br>CATCATGGTTTACATGCTACAGTCAAGATGCGAATCATTATTTGCTGCTCTAG<br>AAATTTAAGGAAATTCATTCAAAACTATGTTTTCATCATCAGATGTTCGTTTTA<br>TGTTTGGATGAACTGACATACTTGTTCCACTCTAGCAGCACGTAAATATTGGC<br>GTAGTGAAATATATATTAAACACCAATATTACTGTGCTGCTTTAGTGTGACAG<br>GGATACAGCAAC**GGATCCC**
hsa-miR-16	ID: 000391	185 bp	**GAATTC**CTTCTGAAGAAAATATATTTCTTTTTATTCATAGCTCTTATGATAGCA<br>ATGTCAGCAGTGCCTTAGCAGCACGTAAATATTGGCGTTAAGATTCTAAAATT<br>ATCTCCAGTATTAACTGTGCTGCTGAAGTAAGGTTGACCATACTCTACAGTTG<br>TGTTTTAATGTATATTAATGTTACTAATGTG**GGATCCC**
hsa-miR-195	ID: 000494	188 bp	**GAATTC**AGTAAGTTCTGCCTCAAGAGAACAAAGTGGAGTCTTTGTTGCCCAC<br>ACCCAGCTTCCCTGGCTCTAGCAGCACAGAAATATTGGCACAGGGAAGCGA<br>GTCTGCCAATATTGGCTGTGCTGCTCCAGGCAGGGTGGTGAAAACTACCGA<br>GGAGGGGCTGAGCCCCCATGGGCCGAGGAGAGAAGAGGGA**GGATCCC**
hsa-miR-424	ID: 000604	432 bp	**GAATTC**TTTCGGCTCCACCTGCAGCTCCTGGAAATCAAATGGTGAAGTTAT<br>AAGAAGTACCTTAACTTGGAGTGAAGTGGCCTAGTCATAAGTTTTGCCCTA<br>TTCCTTGCCAGGGGGTGGGGCGGGGCTTCCTTCAGTCATCCAGTCTTTAT<br>TCACCCGCAGGTACCCCCAGATCGATCCCCCTTCATTGACTCCGAGGGGA<br>TACAGCAGCAATTCATGTTTTGAAGTGTTCTAAATGGTTCAAAACGTGAGG<br>CGCTGCTATACCCCCTCGTGGGGAAGGTAGAAGGTGGGGTCTGCCGGAC<br>GCGTGTTCCTGCCACCAGGTGCCCGCTCCCCGCGAGGCCGGCTCAGGAG<br>CAGGTAGGTGGGCGGGGGCTCGCGTCTCTGTTTCCGGCGGGTCCTCGCC<br>CTTGTCCACGCAGCAGGGCTGTAGTCCGGGGAGGGCAG**GGATCCC**
hsa-miR-497	ID: 001043	212 bp	**GAATTC**GGTGGTGCTGGGGTCTTCCCAGCACTGCTATGTGCTCTCTTCCT<br>TTCAACCCACCCCGGTCCTGCTCCCGCCCCAGCAGCACACTGTGGTTTGT<br>ACGGCACTGTGGCCACGTCCAAACCACACTGTGGTGTTAGAGCGAGGGTG<br>GGGGAGGCACCGCCGAGGCTTGGCCCTGGGAGGCCATCCTGGAGAAGTG<br>ACACAAAAAACATCTGGGG**GATCCC**
hsa-miR-6838	ID: 467053_mat	156 bp	**GAATTC**GAGCACACCCCACCTCGGCGCTGGGCTCTTCCCCACCCTCAGTC<br>TTCCTGCAGGGAAGCAGCAGTGGCAAGACTCCTAGGTCACGGAAGTCCTG<br>CTTCTGTTGCAGAAGACATTTTTCCAAGCGGCTTCAGAGGAAGACATCTTC<br>AGACACCTGGG**GGATCCC**

Supplementary Table 4: Correlation analysis between the expression level of miRNAs, SPAG7, and basic semen parameters.

MicroRNA	Parameters	Count (10^6/ml)	Motility (% motile)	Morphology (%)	SPAG7
**miR-15a-5p**	Correlation Coefficient	0.274	0.328	0.105	0.133
	P value	0.063	0.011	0.425	0.310
**miR-15b-5p**	Correlation Coefficient	0.387	0.400	0.116	0.175
	P value	0.001	0.001	0.337	0.146
**miR-16-5p**	Correlation Coefficient	0.444	0.302	0.079	0.372
	P value	<0.0001	0.011	0.516	0.002
**miR-195-5p**	Correlation Coefficient	-0.152	-0.244	-0.063	-0.208
	P value	0.212	0.044	0.608	0.087
**miR-424-5p**	Correlation Coefficient	-0.526	-0.602	-0.274	-0.406
	P value	<0.0001	<0.0001	0.024	0.001
**miR-497-5p**	Correlation Coefficient	-0.233	-0.299	0.032	-0.293
	P value	0.052	0.012	0.793	0.014
**miR-6838-5p**	Correlation Coefficient	-0.150	-0.165	-0.070	-0.063
	P value	0.236	0.192	0.583	0.619

- Spearman correlation analysis
- An Unpaired two-tailed t-test was used to calculate the p-values.
- $P < 0.05$ was considered statistically significant.
